# Transcript-Specific Expression Profiles Derived from Sequence-Based Analysis of Standard Microarrays

**DOI:** 10.1371/journal.pone.0004702

**Published:** 2009-03-11

**Authors:** Anton G. Moll, Maja T. Lindenmeyer, Matthias Kretzler, Peter J. Nelson, Ralf Zimmer, Clemens D. Cohen

**Affiliations:** 1 Institute of Physiology and Clinic for Nephrology, University of Zürich, Zürich, Switzerland; 2 Medizinische Poliklinik, Klinische Biochemie, Ludwig-Maximilians-Universität München, München, Germany; 3 Center for Computation in Medicine and Biology, University of Michigan, Ann Arbor, Michigan, United States of America; 4 Institut für Informatik, Ludwig-Maximilians-Universität München, München, Germany; University of the Western Cape, South Africa

## Abstract

**Background:**

Alternative mRNA processing mechanisms lead to multiple transcripts (i.e. splice isoforms) of a given gene which may have distinct biological functions. Microarrays like Affymetrix GeneChips measure mRNA expression of genes using sets of nucleotide probes. Until recently probe sets were not designed for transcript specificity. Nevertheless, the re-analysis of established microarray data using newly defined transcript-specific probe sets may provide information about expression levels of specific transcripts.

**Methodology/Principal Findings:**

In the present study alignment of probe sequences of the Affymetrix microarray HG-U133A with Ensembl transcript sequences was performed to define transcript-specific probe sets. Out of a total of 247,965 perfect match probes, 95,008 were designated “transcript-specific”, i.e. showing complete sequence alignment, no cross-hybridization, and transcript-, not only gene-specificity. These probes were grouped into 7,941 transcript-specific probe sets and 15,619 gene-specific probe sets, respectively. The former were used to differentiate 445 alternative transcripts of 215 genes. For selected transcripts, predicted by this analysis to be differentially expressed in the human kidney, confirmatory real-time RT-PCR experiments were performed. First, the expression of two specific transcripts of the genes PPM1A (PP2CA_HUMAN and P35813) and PLG (PLMN_HUMAN and Q5TEH5) in human kidneys was determined by the transcript-specific array analysis and confirmed by real-time RT-PCR. Secondly, disease-specific differential expression of single transcripts of PLG and ABCA1 (ABCA1_HUMAN and Q5VYS0_HUMAN) was computed from the available array data sets and confirmed by transcript-specific real-time RT-PCR.

**Conclusions:**

Transcript-specific analysis of microarray experiments can be employed to study gene-regulation on the transcript level using conventional microarray data. In this study, predictions based on sufficient probe set size and fold-change are confirmed by independent means.

## Introduction

DNA microarrays are important experimental tools to gain knowledge about the steady state levels of mRNA species. Affymetrix GeneChips were designed to contain a series of oligonucleotide probes complementary to a specific mRNA of known genes. To quantify a specific mRNA species, the signals from a group of probes (i.e. probe sets) representing a specific gene are normalized and averaged (e.g. [1]). However, often the design of the array and the selection of probe sequences were finalized before the human genome was fully annotated. Therefore some probes lack specificity and the conventional probe sets do not always reflect current knowledge about the multiple individual transcripts encoded by the same gene. Furthermore, mRNA processing mechanisms can lead to different transcripts of the same gene which can have specific biological functions [1], [2]. Methods that apply microarray profiling would – by using the available and measured transcript-specific probes – provide additional information not on the expression levels of a gene but also the respective splice isoforms.

Several tools have been developed to customize the analysis of gene expression data. Novel mapping of given probe sequences to more recent genomic data was performed by Gautier et al. [3] and Harbig et al. [4]. Both groups were able to show that re-mapping of the probe sequences affects data analysis for specific arrays. Dai et al. [5] introduced re-defined probe sets after re-alignments of probe sequences to genes as well as transcripts. However, as probes of a given probe set were allowed to match several different transcripts, their overall signal will still be influenced by several transcripts. Additional alignment algorithms were used to define transcript-specific probe sets employing different databases as RefSeq or AceView [6], [7]. However, none of these reports experimentally validated alternative transcript expression.

Independent confirmatory experiments by real-time RT-PCR or other techniques are clearly advisable for any microarray results obtained with a modified measurement or analysis protocol [8]. The goal of the present work was to annotate probe sequences of the widely employed microarray Affymetrix HG-U133A to Ensembl transcript definitions and select probes whose sequences are specific and suitable to estimate expression of transcripts. Re-defined transcript-specific probe sets were used on available microarray data of human renal tissue [9] to predict the expression of individual transcripts. Two potential biological differences in the expression profile of transcripts were analyzed: First, genes having a lowly as well as a highly abundant transcript in healthy human renal tissue were searched. Secondly, genes were selected with differentially regulated transcripts between normal renal tissue and tissue from kidney patients. From both analyses, two genes were selected for confirmatory experiments and their respective transcript expression levels quantified using transcript-specific real-time RT-PCR on the identical mRNA used for array hybridization.

## Results

### Defining transcript- and gene-specific probe sequences

To determine transcript-specific expression information from the HG-U133A microarray, individual probe sequences were first aligned to Ensembl transcript sequences, then grouped to transcript-specific probe sets, which were employed in robust multiarray average analysis (RMA, see Methods).

In the Blast analysis two types of alignments were exploited: “exact alignment”, i.e. 100% sequence identity over 25 base pairs of transcript and probe sequence, and “near-exact alignment” with at most one mismatch (identity of 24 base pairs). We defined transcript-specific probes to have one “exact alignment” with a transcript, but no “exact alignment” in other transcripts, and no “near-exact alignment”. For gene-specific probes the same definition was used with respect to alignments to transcript sequences of one gene (instead of alignment to one transcript). Probes showing evidence for cross-hybridization (“exact alignments” to transcript sequences of more than one gene) were excluded from this study. The alignment of probes to Ensembl transcripts gave 95,008 transcript-specific and 180,403 gene-specific probes out of a total of 247,965 probes (**Table 1** **, supporting** **tables S1** **and** **S2**, and **dataset S1**).

**Table 1:** Transcript-specificity of HG-U133A probes to Ensembl transcripts.

number of probes	number of transcripts
56388	0
95008	1
50011	2
19901	3
9836	4
5131	5
2895	6
1721	7
…	…
1	44
1	50

The number of probes with at least one “exact alignment” with a transcript, and no “near-exact alignments” were computed and grouped by the number of different transcripts they have an “exact alignment” with. The probes in this table with number of transcripts equal to 1 are the transcript-specific probes.

**Figure 1:**
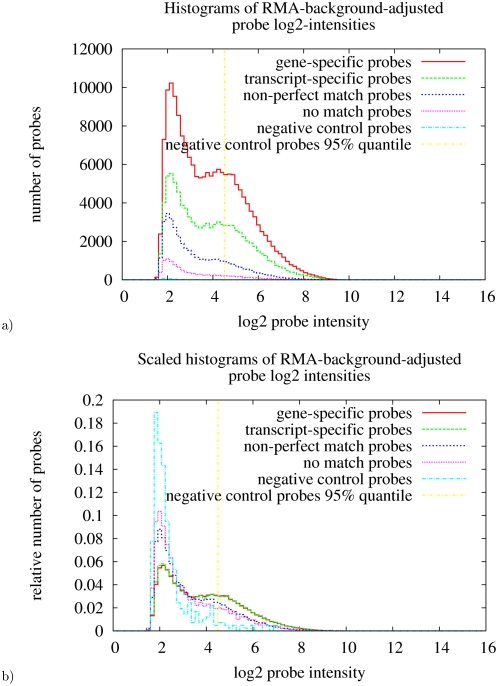
Intensity histograms of several probe categories. Gene-specific and transcript-specific probes show more high intensity signals as compared to non-perfect or no-match probes. a) Probe intensities of one microarray (LD2) were RMA-background-adjusted and logarithmized (base 2). Probes were assigned to categories shown in the legend as defined in the results section. The normalized intensities of each category are put into 100 evenly-spaced intervals and plotted as histogram. The probe intensity 95%-quantile of Affymetrix-defined negative control probe sets is plotted in yellow. b) As the number of probes varies between the different categories it is hard to compare the shapes of the distributions in a). Therefore, the histograms were normalized with their number of probes in this plot. Averaging over multiple arrays instead of analyzing one array gives similar results.

To roughly test the characteristics of the transcript- and gene-specific probes, five probe categories were defined: transcript-specific, gene-specific (both as defined above), non-perfect match probes (alignments with gaps or mismatches), no-match probes (no alignment with an e-value smaller than 1), and negative control probes (non-human sequence, indicated by the manufacturer). Probes with transcript- or gene-specific alignments were expected to show higher signal intensities in an experiment due to more complete template hybridization compared to non-perfect match, no-match, or negative control probes. This was tested on data from human renal tissue. Signal intensity histograms of probes of the above categories were plotted from a single array experiment (**Figure 1**). Non-perfect match probes had, as expected, a similar intensity distribution as probes with no match. Gene-specific probes showed an almost identical distribution compared to transcript-specific probes. These specific probes had a higher percentage of probes with high intensity signals compared to non-perfect or no match probes: Choosing the 95%-quantile of the intensities of non-human control probes a higher fraction of gene- and transcript-specific probes showed intensities above this threshold than non-perfect or no-match probes (33.8%, 33.7%, 21.1%, 18.8%; respectively). The specific probes showed higher signal intensities than non-specific probes probably due to complete hybridization with corresponding templates. A few non-specific probes showed also high signal intensities potentially corresponding to as of yet unidentified transcripts (in Ensembl).

Grouping such individual probes to probe sets resulted in 7,941 transcript-specific probe sets and 15,619 gene-specific probe sets. The general size of probe sets were multiples of 11, since the original HG-U133A array probe sets consist of 11 probes each. The transcript-specific probe sets were then “re-annotated” to the respective gene to determine how many individual transcripts could be quantified for a specific gene. For 215 genes, two or more transcripts were covered by the newly defined transcript-specific probe sets with a minimal probe set size of 1 (**Table 2**). A minimal probe set size of 3 reduced the number of genes to 141 (136 genes with 2 transcripts, 4 genes with 3 transcripts, 1 gene with 4 transcripts).

**Table 2:** Genes having transcripts with transcript-specific probes.

number of known transcripts	genes with 1 matched transcript	genes with 2 matched transcripts	genes with 3 matched transcripts	genes with 4 matched transcripts	genes with 5 matched transcripts
1	5447				
2	1162	99			
3	480	49	2		
4	192	25	2	1	
5	95	11	3		
6	54	2	1		
7	27	5			
8	16	3			
9	9	4			
10	4	1		1	1
11	4	2			
12	1				
13	1				
14	1				
15	1				
16	1				
17		1			
18	1				
sum	7496	204	8	2	1

The fraction of transcripts that were matched by transcript-specific probes, listed by total number of known transcripts in Ensembl, are shown. For example, 99 genes had 2 known transcripts and both had at least one transcript-specific probe, while 1,162 had a transcript-specific probe set (of size> = 1) for only one transcript.

The newly defined probe sets were then used to analyze human renal gene expression data to predict transcript-specific expression profiles further to be confirmed by real-time RT-PCR.

### Analysis of high and low abundant renal mRNA transcripts

**Figure 2:**
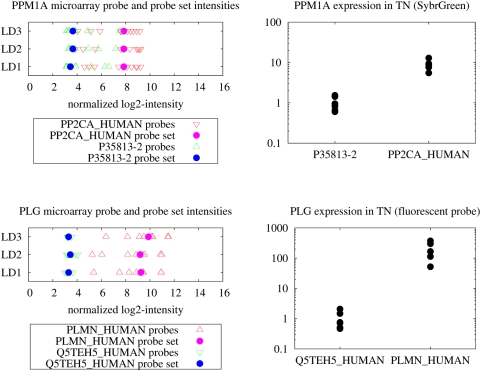
Comparison of alternative transcript abundance in microarray and real-time RT-PCR. On the left microarray signal intensities are shown for the genes PPM1A and PLG. Confirmatory real-time RT-PCR data are shown on the right. Lowly and highly abundant transcripts of the genes PPM1A and PLG were measured using microarrays in LD tissue. Transcript-specific probe intensities were background-adjusted and quantile-normalized using RMA. These are shown as single probe values (triangles) and, furthermore, as summarized transcript-specific probe set values (dots). In addition, the transcripts were quantified in the unaffected part of TN tissue using real-time RT-PCR. Real-time RT-PCR data are normalized to the transcript with lower abundance.

To test the alignment data and to identify genes with differential expression of alternative transcripts in human renal tissue, transcript levels for two genes were first tested based on the newly defined transcript-specific probe sets. Expression levels were then validated in a confirmatory experiment by real-time RT-PCR using the same cDNA hybridized on the array. For this experiment four transcripts of two genes were selected from expression data of human kidneys (living allograft donors (LD); n = 3) with the following rationale: The distributions of transcript-specific probe intensities appeared distinguishable (no or little overlap of probe intensities between the transcripts) and the number of probes for each transcript was at least 3. For both selected genes, PPM1A and PLG, two transcripts have been annotated in Ensembl and for each transcript, 6 to 11 specific probes are available on the array. In the case of gene PPM1A higher signal levels of the probe set for transcript PP2CA_HUMAN were observed than of the probe set for the second transcript P35813-2 (7.8±0 and 3.6±0.1, respectively; p<0.001). For the gene PLG the probe set for transcript PLMN_HUMAN showed higher signal intensities than the one for the transcript Q5TEH5_HUMAN (9.4±0.4 and 3.4±0.1, respectively; p<0.01) (see **Figure 2**).

Real-time RT-PCR quantification of the transcripts on independent kidney samples (tumor nephrectomies (TN); n = 6) gave transcript-specific expression values in agreement with the transcript-specific expression pattern generated with the microarray expression data (PPM1A: PP2CA_HUMAN: 8.7±2.5; P35813-2: 1±0.4, p<0.001; PLG: PLMN_HUMAN 217.8±125.8; Q5TEH5_HUMAN 1±0.6, p<0.01) (see **Figure 2**). This “qualitative” approach – although problematic due to comparison of signal intensities of different microarray probes which have different hybridization efficiencies (see e.g. [10]) – underlined the prospects of a transcript-specific analysis, which were further studied on the quantitative level as follows.

### Analysis of quantitative differences in renal mRNA transcripts

**Figure 3:**
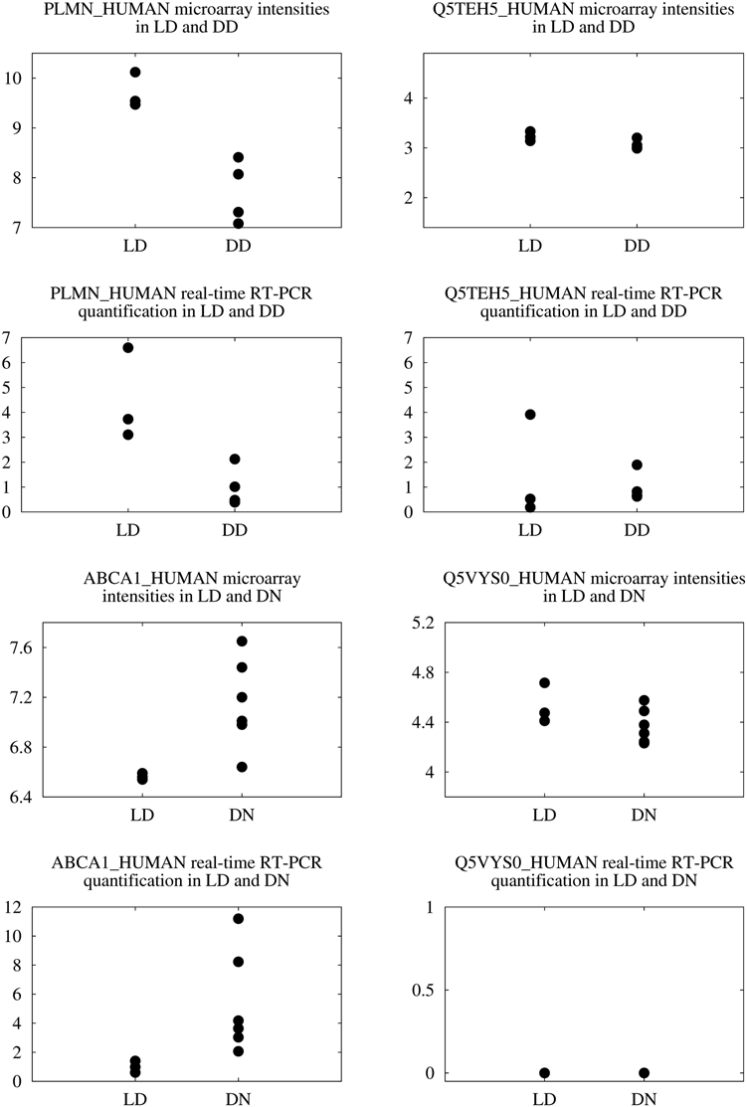
Microarray and real-time RT-PCR transcript measurements of PLG and ABCA1 in two patient cohorts. For the gene PLG, transcript PLMN_HUMAN was repressed in DD compared to LD controls, while Q5TEH_HUMAN showed no differential expression. Real-time RT-PCR measurements on the same tissues were in agreement with these findings. For gene ABCA1, transcript ABCA1_HUMAN was induced in DN compared to LD, and Q5VYS0_HUMAN was not regulated. Real-time RT-PCR measurement confirmed the induction of ABCA1_HUMAN. Q5VYS0_HUMAN was expressed at a too low level to be measured (value 0). Real-time data are normalized to the cohort with lower abundance.

To find genes that showed transcript-specific alterations between different patient cohorts, two analyses were performed: Expression profiles from renal allografts from LD (n = 3) were compared to renal biopsies from deceased (cadaveric) allograft donors (DD; n = 4) and the same LD expression data were compared to renal biopsies from patients with diabetic nephropathy (DN; n = 10). After background-correction, quantile normalization and summarization according to transcript-specific probe set definition (see methods) these data were analyzed for fold changes and q-values using significance analysis of microarrays (SAM) [11]. Genes with i) a quotient of maximal to minimal transcript-specific fold change of at least 1.5 and ii) at least one transcript with q-value<0.1 between LD vs. DD or LD vs. DN, respectively, were considered for experimental confirmation. Nine genes for LD versus DD and four genes for LD versus DN passed these filter criteria. Two differentially expressed transcripts from two genes were selected based on probe set size consideration. These isoforms had at least six probes per probe set. Transcript PLMN_HUMAN of the PLG gene showed reduced expression in DD compared to LD while transcript Q5TEH5_HUMAN of the same gene was not altered in expression. For gene ABCA1, transcript ABCA1_HUMAN was induced in DN compared to LD and transcript Q5VYS0_HUMAN was not changed in DN (see **Table 3** and **Figure 3**).

**Table 3:** Significance analysis of intensity differences between alternative transcripts of two genes.

gene	transcript	SAM fold change	SAM q-value	probe set size
PLG	PLMN_HUMAN	0.264 (LD vs. DD)	0.057	9
	Q5TEH5_HUMAN	0.888 (LD vs. DD)	0.182	6
ABCA1	ABCA1_HUMAN	1.516 (LD vs. DN)	0.053	16
	Q5VYS0_HUMAN	0.891 (LD vs. DN)	0.326	10

The microarray intensities for transcript-specific probe sets of gene PLG (ABCA1) were compared between LD and DD (DN). Fold change indicates the induction or repression of the respective transcript compared to the healthy LD controls.

The transcripts selected were again quantified using real-time RT-PCR. Results are displayed in **Figure 3** and confirm a concordant differential expression compared to microarray data for PLG for both transcripts (PLMN_HUMAN: LD 1±0.4; DD 0.2±0.2, p<0.05; Q5TEH5_HUMAN: LD 1±1.3; DD 0.7±0.4, n.s.). The induction of the transcript ABCA1_HUMAN in DN observed in the microarray data was also confirmed by real-time RT-PCR (LD 1±0.4, DN 5.4±3.5, p<0.05). The signals for Q5VYS0 in real-time RT-PCR were too low to be quantified probably due to low mRNA expression.

## Discussion

Default annotation of first and second generation Affymetrix microarrays such as HG-U133A is not compatible with up-to-date transcript information and does not allow the selective analysis of specific transcripts. The latest generations of microarrays, like exon arrays or tiling arrays, address transcript expression analysis by an increased number of probes and respective selection of probe sequences. However, large sets of data have been generated and are being generated with the earlier microarray generations. Thus, re-analysis of these data could provide more detailed information of transcript expression using transcript-specific probe sets.

This approach was employed by aligning the probe sequences of the Affymetrix Array HG-U133A to the Ensembl transcript sequences and selecting transcript-specific probes to build transcript-specific probe sets. We found that the probe sequences on the HG-U133A were sufficient to distinguish multiple transcript intensities for 215 genes (or 141 genes with a minimum probe set size of 3). In our proof-of-concept application, four selected transcripts with different expression levels in the kidney were identified and renal expression confirmed by real-time RT-PCR. As the intensity of a probe depends not only on the concentration of its complementary transcript but also on its sequence, such an approach may be problematic. Therefore, disease-associated and transcript-specific differential expression was also studied. Again, both predicted expression patterns were supported by real-time RT-PCR experiments.

Different groups reported recently their approaches to map probe sequences of Affymetrix microarrays to transcript sequences. Some of these mention transcript-specific analysis as a possible application, however, a confirmation of a predicted transcript expression by independent means such as real-time RT-PCR has not yet been reported. Dai et al. [5] aligned probe sequences of several microarrays to transcript sequences from different databases and derived “transcript-specific” probe sets. However, as the authors noted, this implies redundancies in related probe set definitions, such as shared probes among different transcripts from the same gene. The intensities of the transcript-specific probes may be lost for the intensity of the probe set. Liu et al. provided AffyProbeMiner [6], which aligns probe sequences to RefSeq (or RefSeq plus GenBank) complete coding sequences. It allows users to choose parameters how probe sets are built. One allowed combination of parameters, in their paper called “transcript-unique probe sets”, is similar to the probe set definition used in our present study. However, we excluded probes having an alignment with one mismatch and used Ensembl instead of RefSeq (plus GenBank). AffyProbeMiner defines 10,226 (6,878 for RefSeq plus GenBank) probe sets, whereas our approach yields 7,941 probe sets (of these 3,412 (1,776) probe sets are identical between the two approaches). Lu et al. [7] also reported a similar approach but using AceView as the reference database. They could show that cross-platform comparability is improved when the transcriptome is analyzed by a transcript-specific approach and a minimum probe set size of four is used. Our group recently reported a study showing improved elucidation of biological processes by single-probe analysis. In this study a commercially available software, ChipInspector, was used, which also employs a re-annotation of probe sequences but uses in house annotation and does not employ probe set definition [12].

Beside these transcript-specific re-annotation approaches an exon-based analysis would be a promising strategy. Such an exon-, instead of transcript-based analysis would offer the advantage of less transcript annotation changes. But applying this approach to routine arrays such as the HG-U133A means fewer probes per probe set and would subsequently reduce the statistical power for determining expression changes. Only the latest generation of exon-specific microarrays yield sufficient data for such an approach.

Our study used a re-annotation approach similar to some of the above reports. Other researchers published web-based tools for the mapping of probe sets to known splice isoforms [13], [14] or used different databases like the International Protein Index [15] or GeneAnnot [16].

But none of these studies supported the bioinformatics data by additional experimental validation. In the present study we validated the expression change of four specific transcripts by real-time RT-PCR. These transcripts were selected as a sufficient number of probes for each transcript showed minor overlap in probe intensities. For PLG, the gene for plasminogen, we observed higher overall expression of the transcript PLMN_HUMAN compared to Q5TEH5_HUMAN in the human kidney. Furthermore, the mRNA for the transcript PLMN_HUMAN was reduced in organs from deceased kidney donors compared to living donors. As plasminogen is mainly synthesized in the kidney the results are in agreement with PLMN_HUMAN being the main transcript of the PLG gene and it seems that its expression is rapidly reduced in a deceased organism. Although it is well-established that the regulation of plasminogen activation plays a crucial role in kidney disease [17], it is difficult to speculate on the biological relevance of the transcript-specific findings for PLG as only little is known about the functions of the two PLG transcripts. The finding of the transcript-specific expression pattern of PPM1A, a protein serine/threonine phosphatase capable of dephosphorylating Smad1 to terminate TGFbeta signaling [18], may well have biological relevance as Smad- and TGF beta-related biological processes are crucial for the progression of kidney diseases [19]. But again the knowledge about the functional differences of the specific transcripts is still limited. With respect to the increased renal synthesis of one transcript of ABCA1, coding for a cholesterol efflux pump, observed in DN it is obvious that this may represent the response of the kidney to the metabolic changes in long-standing diabetes mellitus including hypercholesteremia, proteinuria and lipiduria [20].

The examples on PLG, PPM1A, and ABCA1 clearly show that for most transcripts the information about their specific biological functions is still limited. However, employing tools like the one defined above will help to increase our knowledge on transcript-specific regulatory events in human disease and animal models.

Re-annotation of probe sequences of conventional microarrays can be employed to define transcript-specific probe sets. (Re)-analyses of established microarray data by transcript-specific probe set definitions are feasible and can give reliable results. This was exemplarily shown on a, although limited, number of genes and transcripts.

## Methods

### Procurement of RNA Samples

Human renal biopsy specimens and HG-U133A (Affymetrix) microarray expression data thereof were procured in an international multicenter study, the European Renal cDNA Bank - Kroener-Fresenius biopsy bank (ERCB-KFB). The protocol for tissue preparation and mRNA isolation has been reported elsewhere [21]. Diagnostic renal biopsies were obtained from patients after informed consent and with approval of the local ethics committees. The microarray expression data used in this study came from individual diabetic patients with established diabetic nephropathy and renal insufficiency (DN; n = 10) as well as deceased allograft donors (DD; n = 4). Pre-transplantation kidney biopsies from living donors (LD; n = 3) were used as control renal tissue. Microdissected samples taken from the tubulo-interstitial compartment were processed as described [9].

### Probe and Transcript Sequences and BLAST

HG-U133A Affymetrix “perfect match probe” sequences and coordinates (247,965 sequences) were downloaded from the Affymetrix support web page [22]. Transcript sequences (44,676 sequences) were extracted from Ensembl ftp release 42 [23]. Probes were aligned to transcript sequences using BLAST version 2.2.15. Probe, transcript, alignment, probe set and array data were stored in a MySQL 5.0 database.

### Gene- and Transcript-specific Probes and Probe sets

The transcript sequences were put into a local BLAST [24] database using the formatdb program. *blastall* options were “-e 1 -m 8 -p blastn -F F”. Low-complexity filtering of query sequences was turned off in order to find all possible hits. Only alignments with an E-value<1.0 were returned. Blastn parameters for match/mismatch score, gap open and gap extension cost were left at their default settings.

Finding exact alignments between probe and transcript sequences was used to attribute signal intensities of probes to transcript (or gene) expression. Therefore, probes were categorized as “transcript-specific” if their exact hits (100% identity over 25 base pairs) were to one transcript only and “gene-specific” if their exact hits were to (possibly more than one) transcripts of a single gene only. This means that transcript-specific probes are a subset of gene-specific probes. Probes with any single mismatch were excluded. Overall, of the 247,965 probes, 180,403 were gene- and 95,008 were transcript-specific. Only the Affymetrix “perfect match (PM) probes” were analyzed as we found that including the “mismatch (MM) probes” would yield only 16 additional transcript-specific probes.

According to above definition of gene- and transcript-specific probes, these were grouped as gene- or transcript-specific probe sets. For example, two gene-specific probes both matching the same transcripts of the same gene were grouped into the same gene-specific probe set. To select transcripts for independent quantification only the transcript-specific probe set intensities were used for this study. To facilitate microarray analysis using the Bioconductor affy library [25], the transcript-specific probe set definitions were stored in a CDF file using the Bio::Affymetrix::CDF perl module [26].

### Selection of Transcripts for Confirmatory Experiments

For the organ specific analysis, expression data from LD were background corrected with the “rma” method and normalized with the “quantiles” method using the R affy library [25]. Genes were then ordered by an ad-hoc method (fitting the probe intensity histogram with a mixture of two gamma distributions, representing low and high intensities of probes, and computing the relative probability that transcripts are from the first or second distribution). Two genes were manually selected, each with two known isoforms and different intensities.

To find genes that showed two transcripts with different expression between two patient cohorts, microarray expression data were background-corrected and quantile normalized as described above. This was done separately for LD vs. DD and LD vs. DN. The normalized probe intensities were summarized using the generated transcript-specific CDF environment. These data were then analyzed for fold changes and q-values between two different patient cohorts using the “sam” function in the “siggenes” R library [27]. From all genes, we extracted the ones with differential expression of specific transcripts. Only genes with at least two transcript-specific probe sets, with at least one transcript having a q-value<0.1 and with a quotient of the maximal/minimal transcript fold change greater than 1.5 were considered for independent quantification. Additionally, we avoided selecting genes with less than three probes per transcript-specific probe set.

### Real-Time RT-PCR Confirmatory Experiments

For validation of the microarray data, real-time RT-PCR transcript quantification analyses were performed on cDNA used in the microarray experiments as well as on samples from the unaffected part of tumor nephrectomies (TN; n = 6).

Real-time RT-PCR was performed on an ABI PRISM 7700 Sequence Detection System (“TaqMan”, Applied Biosystems, Darmstadt, Germany) using heat-activated TaqDNA polymerase (Amplitaq Gold; Applied Biosystems). After an initial hold of two minutes at 50°C and ten minutes at 95°C, the samples were cycled at 95°C for 15 seconds and 60°C for 60 seconds. For normalization, commercially available pre-developed TaqMan reagents were used for the housekeeper gene 18S rRNA (Applied Biosystems). Oligonucleotide primers (300 nmol/L) and probe (100 nmol/L) used are listed in **Table 4**. All primers, including the primers for Q5VYS0, gave positive signals on whole kidney mRNA. Water and no template controls were negative (Ct>40).

**Table 4:** Primers used for real-time RT-PCR.

gene	transcript	primer direction	position	primer length	primer sequence	product size
PPM1A	P35813-2	forward	1274	24	CCTGTTTGTATAAGGGAAGTCGAG	195
		reverse	1468	27	AAGTTTGATTGTGTTGAAGATTTTTCT	
	PP2CA_HUMAN	forward	1136	24	CCTGTTTGTATAAGGGAAGTCGAG	248
		reverse	1384	20	CATTCCTCTTGCTTGCCAAT	
PLG	Q5TEH5_HUMAN	forward	1015	24	GAGTTTTAGGCCAAATCTGAGAAA	109
		probe	1043	33	CAAAGATGACTATGTTTGGGACTGAAGTAAGCA	
		reverse	1123	20	TTGCTCCACAATTTGAGTCG	
	PLMN_HUMAN	forward	905	20	AAAACTATCGCGGGAATGTG	110
		probe	956	25	ACTGGAGTGCACAGACCCCTCACAC	
		reverse	1014	20	TTTGCAGGGGAAGTTTTCTG	
ABCA1	ABCA1_HUMAN	forward	1670	21	CTTCATGGAGAACAGCCAAGA	76
		probe	1691	25	AATGGACCTTGTCCGGATGCTGTTG	
		reverse	1745	21	TTCCCAAAAGTGGTCATTGTC	
	Q5VYS0_HUMAN	forward	990	21	AGCGAGTACTTCGTTCCAACA	77
		probe	1018	25	CCTGAAGCCAATCCTGATGGATGTG	
		reverse	1066	19	CCCATGTGCAATGTCATCA	

These primers were used for quantification of highly and lowly abundant renal mRNA transcripts and difference between renal mRNA transcripts. PPM1A transcripts' intensities were measured using SYBR Green, while those of PLG and ABCA1 were measured using an internal fluorescent probe.

### Statistics

Significance testing (one-sided t-test) results were considered to be significant for p-values smaller than 5%. They were computed using Gnumeric version 1.6.3 [28]. The SAM q-value can be interpreted analogous to a p-value that is corrected for multiple testing.

## Supporting Information

Table S1 List of Ensembl transcript identifiers of the 7,941 transcripts covered by transcript-specific probe sets.
(0.13 MB TXT)

Table S2 List of genes with multiple transcripts covered by the defined transcript-specific probe sets. For 215 genes, two or more transcripts were covered by transcript-specific probe sets with a minimal probe set size of 1. This table lists those genes along with the covered 445 transcripts.
(0.01 MB TXT)

Dataset S1 CDF file describing the transcript-specific probe sets. The transcript-specific probe set definitions were stored in a CDF file using the Bio::Affymetrix::CDF perl module. For use in some analysis tools it may be necessary to rename this CDF file to the default Affymetrix file name (“HG-U133A.CDF”).
(2.87 MB ZIP)
